# The Oncogenic Protein Kinase/ATPase RIOK1 Is Up-Regulated via the c-*myc*/E2F Transcription Factor Axis in Prostate Cancer

**DOI:** 10.1016/j.ajpath.2023.05.013

**Published:** 2023-06-09

**Authors:** Florian Handle, Martin Puhr, Martina Gruber, Chiara Andolfi, Georg Schäfer, Helmut Klocker, Johannes Haybaeck, Peter De Wulf, Zoran Culig

**Affiliations:** ∗ Department of Urology, Medical University of Innsbruck, Innsbruck, Austria; † Institute of Pathology, Neuropathology and Molecular Pathology, Medical University of Innsbruck, Innsbruck, Austria; ‡ Diagnostic and Research Center for Molecular Biomedicine, Institute of Pathology, Medical University of Graz, Graz, Austria; § Department of Cellular, Computational, and Integrative Biology (CIBIO), University of Trento, Trento, Italy

## Abstract

The atypical protein kinase/ATPase RIO kinase (RIOK)-1 is involved in pre-40S ribosomal subunit production, cell-cycle progression, and protein arginine *N*-methyltransferase 5 methylosome substrate recruitment. RIOK1 overexpression is a characteristic of several malignancies and is correlated with cancer stage, therapy resistance, poor patient survival, and other prognostic factors. However, its role in prostate cancer (PCa) is unknown. In this study, the expression, regulation, and therapeutic potential of RIOK1 in PCa were examined. RIOK1 mRNA and protein expression were elevated in PCa tissue samples and correlated with proliferative and protein homeostasis-related pathways. *RIOK1* was identified as a downstream target gene of the c-*myc*/E2F transcription factors. Proliferation of PCa cells was significantly reduced with RIOK1 knockdown and overexpression of the dominant-negative RIOK1-D324A mutant. Biochemical inhibition of RIOK1 with toyocamycin led to strong antiproliferative effects in androgen receptor–negative and –positive PCa cell lines with EC_50_ values of 3.5 to 8.8 nmol/L. Rapid decreases in RIOK1 protein expression and total rRNA content, and a shift in the 28S/18S rRNA ratio, were found with toyocamycin treatment. Apoptosis was induced with toyocamycin treatment at a level similar to that with the chemotherapeutic drug docetaxel used in clinical practice. In summary, the current study indicates that *RIOK1* is a part of the *MYC* oncogene network, and as such, could be considered for future treatment of patients with PCa.

Prostate cancer (PCa) is among the most frequently diagnosed cancers in men, with nearly 1.5 million new cases recognized worldwide per year.^1^ PCa can be treated by radical prostatectomy and radiation therapy when detected in the early stages.^2^ Several new androgen receptor (AR)–signaling inhibitors, poly–ADP ribose polymerase (PARP) inhibitors, and radio ligand therapies are approved for the treatment of patients with advanced PCa.^3^ However, curative treatment of patients with advanced PCa remains elusive, and all currently available treatment regimens eventually lead to the development of therapy resistance. This demonstrates the urgent need for the identification of novel drug targets to expand the therapeutic opportunities for patients with advanced PCa.

Protein kinases have a pivotal role in cancer initiation and progression. Consequently, >50 clinically approved kinase inhibitors are commercially available.^4^ Several clinical trials of the efficacy of protein kinase inhibitors in the treatment of PCa are ongoing, but have not yet led to clinical approval.^5^ Thus, it is important to investigate still-unexplored protein kinases that promote PCa. Atypical protein kinases do not share clear sequence similarity with conventional kinases but still have protein kinase activity.^4^ RIO kinase (RIOK)-1 is an atypical protein kinase/ATPase that has recently attracted significant interest in the cancer-research community due to its role in several important cellular processes. RIOK1 is involved in the final steps of pre-40S ribosomal maturation, cell-cycle progression, and protein arginine *N*-methyltransferase (PRMT)-5 methylosome substrate recruitment.^6^, ^7^, ^8^ Furthermore, pan-cancer screens have indicated that RIOK1 is up-regulated in many cancer entities,^9^ including lung, breast, and colorectal cancers as well as glioma.^10^, ^11^, ^12^, ^13^ In addition, RIOK1 overexpression is correlated with tumor stage, therapy resistance, poor survival, and other prognostic risk factors in these malignancies.^10^, ^11^, ^12^, ^13^, ^14^, ^15^

Knockdown of RIOK1 leads to reductions in proliferation, migration/invasion, colony formation capacity, and metastasis formation in a variety of cancer cell lines^11^^,^^12^^,^^14^ and mouse xenograft models.^14^ RIOK1 knockdown disrupts AKT signaling and induce p53 activity via the ribosomal protein (RP)-L11–dependent ribosomal stress checkpoint in glioblastoma cells.^16^ Similarly, RIOK1 down-regulates p53 protein stability in colorectal cancer cells.^15^

However, the role of RIOK1 in prostate carcinogenesis has not yet been studied. This study investigated the expression, regulation, and therapeutic potential of RIOK1 in PCa.

## Materials and Methods

### Cell Culture

PC3, LNCaP, and 22Rv1 cells were cultivated in RPMI 1640, and DU145 cells were cultivated in Dulbecco’s modified Eagle’s medium. The media were supplemented with 10% fetal calf serum (catalog number P30-3031; PAN Biotech, Aidenbach, Germany), 1% penicillin/streptomycin (catalog number DE17-602E; Lonza, Basel, Switzerland) and 1× GlutaMAX (catalog number 35050-038; Thermo Fisher Scientific, Waltham, MA). All cells were obtained from ATCC (Manassas, VA), authenticated by short tandem repeat analysis, and regularly checked for mycoplasma infection. Multiwell plates for LNCaP cells were coated with poly-d-lysine (catalog number P6407; Merck Millipore, Burlington, MA). PC3-rtTAM2 cells expressing the Tet-On transactivator were generated by lentiviral transduction, and induction was performed with 500 ng/mL doxycycline.

Transient knockdown experiments were performed by transfection of siRNA at 5.3 pmol/cm^2^ growth area with Lipofectamine RNAiMAX (catalog number 13778150; Thermo Fisher Scientific) using siCtrl, siRIOK1, and siMYC nontargeting ON-TARGETplus SMARTPool siRNAs (catalog numbers D-001810-10-05, L-005368-00-0005, and L-003282-02-0005, respectively; Horizon Discovery, Waterbeach, UK). Transient overexpression was performed in PC3-rtTAM2 cells by transfection of plasmid DNA at 200 ng/cm^2^ with X-tremeGENE HP (catalog number 6366244001; Merck Millipore). Stable knockdown was performed by lentiviral transduction of PC3-rtTAM2 cells with a vector encoding doxycycline-inducible *ZIM3-KRAB-dCAS9* (recloned from Addgene plasmid 154472)^17^ and the following single-guide (sg)-RNAs (sgRNA tracr-v2 expression cassette recloned from Addgene plasmid 96925)^18^: sgCtrl (5′-TTTTACCTTGTTCACATGGA-3′) and sgRIOK1 (5′-TGGCAGGGTGGTGGATCTGT-3′).

Biochemical inhibition of RIOK1 was performed by treatment of the cells with toyocamycin (catalog number HY-103248; MedChemExpress, Monmouth Junction, NJ). Docetaxel was used as a positive control for apoptosis induction.

### Western Blot Analysis

Protein isolation was performed by sonication in sample buffer (250 mmol/L Tris-HCl, pH 8.5, 2% lithium dodecyl sulfate, 10% glycerol, and 0.5 mmol/L EDTA). Protein concentration was determined with bicinchoninic acid (catalog number 23225; Thermo Fisher Scientific). Separation was performed on Bolt 4% to 12% Bis–Tris gels (catalog number NW04122BOX; Thermo Fisher Scientific) and transferred to nitrocellulose membranes (catalog number 10600001; GE Healthcare, Chicago, IL). Blocking and antibody incubation were performed in 5% bovine serum albumin in Tris-buffered saline + 0.05% Tween-20. The following antibodies were used: glyceraldehyde phosphate dehydrogenase (GAPDH) (catalog number MAB374; Merck Millipore), RIOK1 (catalog number ab88496; Abcam, Cambridge, UK), c-*myc* (catalog number 5605; Cell Signaling Technologies, Danvers, MA), and cPARP (catalog number 5625; Cell Signaling Technologies). Detection and quantification were performed using an Odyssey CLx near-infrared imager (LI-COR, Lincoln, NE) and Image Studio software version 5.2 (LI-COR). Protein expression was normalized to GAPDH. Statistical analysis was performed in Excel 2016 (Microsoft, Redmond, WA) using the *t*-test, with three biologically independent replicates.

### Real-Time Quantitative RT-PCR

The Extractme Total RNA Kit (catalog number EM09.2-250; Blirt, Gdańsk, Poland), LunaScript RT SuperMix Kit (catalog number E3010L; New England BioLabs, Ipswich, MA), and Luna Universal Probe qPCR Master Mix (catalog number M3004X; New England BioLabs) were used to prepare samples for real-time quantitative RT-PCR on a CXF Connect Real-Time PCR Detection System (Bio-Rad Laboratories, Hercules, CA). Quantification was performed using CFX Maestro software version 2.0 (Bio-Rad). The geometric means of the reference genes *TBP*, *HMBS*, and *RPLP0* were used for normalization. The following primer/probe sets were used: *TBP* (forward, 5′-CACGAACCACGGCACTGATT-3′; reverse, 5′-TTTTCTTGCTGCCAGTCTGGAC-3′; probe, 5′-FAM-TCTTCACTCTTGGCTCCTGTGCACA-BHQ1-3′), *RPLP0* (forward, 5′-TGCCTCATATCCGGGGGAAT-3′; reverse, 5′-GCAGCAGCTGGCACCTTATT-3′; probe, 5′-FAM-ATCAGGGACATGTTGCTGGCC-BHQ1-3′), *HMBS* (catalog number Hs00609297_m1; Thermo Fischer Scientific), and *RIOK1* (catalog number Hs01574133_m1; Thermo Fischer Scientific). Statistical analysis was performed in Excel using the *t*-test, with three biologically independent replicates.

### Population-Doubling Level

The population-doubling level (PDL) was determined with the formula: PDL = 3.32 × [log_10_ (cell number at the end of the incubation time) – log_10_ (cell number at the beginning of the incubation time)] + starting PDL.

The cell number was measured with a Casy cell counter (Schärfe System, Reutlingen, Germany). Statistical comparison of the linear regression slopes was performed in statistical software package R 4.2.0 using the lsmeans package version 2.30-0 (*https://cran.wu.ac.at*).

### Sub-G_1_ Analysis

Cells were fixed in cold 70% ethanol, washed in phosphate-buffered saline, and stained with 50 μg/mL propidium iodide in phosphate-buffered saline. The percentage of sub-G_1_ cells was determined on FACS Calibur (Becton, Dickinson, and Company, Franklin Lakes, NJ).

### Dose–Response Curves

Cells were treated with a dilution series of toyocamycin for 3 days. The effect on cell number was measured by SYBR Green I staining on a Cytation 5 plate reader (Agilent, Santa Clara, CA) upon incubation in lysis buffer (1% Triton X-100, 50 μg/mL proteinase K, 40 mmol/L Tris base, 20 mmol/L acetic acid, 1 mmol/L EDTA) for 30 minutes at 37°C. EC_50_ values were determined in statistical software package R using the DRC package version 3.0-1 (*http://www.r-project.org*). Three biologically independent replicates were evaluated.

### Absolute Cell Number Quantification

Absolute cell number quantification per well was performed via Hoechst33341 nuclear staining and counting with ImageJ software version 1.53q (NIH, Bethesda, MD; *http://imagej.nih.gov/ij*) and the Celena S fluorescent microscope (Logos Biosystems, South Korea). Statistical analysis was performed in Excel using the *t*-test, with three biologically independent replicates.

### Quantification of rRNA Content

Total RNA was isolated, measured on a NanoDrop 2000c spectrophotometer (Thermo Fisher Scientific), and normalized to the absolute cell number. Statistical analysis was performed in Excel using the *t*-test. Profiles of rRNA were measured on a TapeStation 4150 (Agilent) using the RNA ScreenTape (catalog number 5067-5576; Agilent). At least three biologically independent replicates were evaluated.

### Reanalysis of Public Data Sets

Publicly available transcriptome data sets were downloaded from the Gene Expression Omnibus repository (*https://www.ncbi.nlm.nih.gov/geo*; accession numbers GSE62872,^19^ GSE21034,^20^ GSE35988,^21^ and GSE193337^22^). RNA sequencing (seq) transcriptome data from The Cancer Genome Atlas (TCGA) Prostate Adenocarcinoma Project^9^ were downloaded from the NIH-GDC data portal (*https://portal.gdc.cancer.gov/projects/TCGA-PRAD*, last accessed May 30, 2023; accession TCGA-PRAD). RNA-seq transcriptome data from metastatic castration-resistant PCa data collection^23^ were downloaded from the cBioPortal repository (*https://www.cbioportal.org/study/summary?id*=*prad_su2c_2019*, last accessed May 30, 2023; accession prad_su2c_2019). Clustered regularly interspaced short palindromic repeats (CRISPR) knockout gene–dependency data generated by the DepMap Consortium^24^ were downloaded from the project website (*https://depmap.org/portal/download/all/?releasename=DepMap*+*Public*+*22Q2*, last accessed May 30, 2023; accession CRISPR_gene_effect.csv).

Bioinformatics analysis was performed in statistical software package R version 4.2.0 (*https://cran.r-project.org*). Bulk differential gene expression analysis was performed with the following packages: Limma software version 3.52.3 (*https://bioconductor.org/packages/release/bioc/html/limma.html*), MetaVolcanoR software version 1.10.0 (*https://www.bioconductor.org/packages/release/bioc/html/MetaVolcanoR.html*), and GSVA software version 1.44.4 (*http://www.bioconductor.org/packages/release/bioc/html/GSVA.html*), using MSigDB hallmark gene sets version 7.5.1. The Pearson method was used for detecting the correlation between two parameters after the removal of extreme outliers (>1.5× interquartile range below/above the first/third quartiles). Single-cell RNA-seq data sets were analyzed with Seurat software package version 4.2.0 (*https://cran.r-project.org/web/packages/Seurat/index.html*) using the SCTransform pipeline and limited to non–immune-related cells. Filtering of poor-quality cells was performed as previously described.^22^ Differential gene expression analysis was performed with MAST package version 1.22.0 (*https://www.bioconductor.org/packages/release/bioc/html/MAST.html*). Gene expression was visualized using weighted kernel density estimation calculated with Nebulosa software package version 1.6.0 (*https://www.bioconductor.org/packages/release/bioc/html/Nebulosa.html*). Pseudo-bulk cluster summarization was performed with normalization to counts per million. The Spearman correlation was used for co-dependency network generation and visualized using the packages GOSemSim software version 2.22.0 (*https://www.bioconductor.org/packages/release/bioc/html/GOSemSim.html*) and igraph software version 1.3.4 (*https://cran.r-project.org/web/packages/igraph/index.html*). Histone mark and transcription factor occupancy data generated by the ENCODE Consortium^25^ were visualized in the University of California–Santa Cruz genome browser (*https://genome.ucsc.edu/cgi-bin/hgGateway*).

### IHC Analysis

The use of archived tissue material from the Innsbruck PCa biobank was approved by the Ethics Committee of the Medical University of Innsbruck (study number 1072/2018). Written informed consent was obtained from all patients in agreement with statutory provisions. In this study, benign and primary cancer tissue sections from two patients and one tissue microarray containing benign and primary cancer tissue cores from 120 PCa patients who underwent radical prostatectomy were used to evaluate RIOK1 protein expression. Staining was performed using the specific antibody RIOK1 (catalog number ab88496; Abcam) at a final dilution of 1:200. Images were taken with a Zeiss Imager Z2 microscope (Carl Zeiss, Oberkochen, Germany) equipped with a Pixelink PL-B622-CU camera (Canimpex Enterprises, Coquitlam, BC, Canada). Tissue microarray images were evaluated using the following modified quick-score protocol: staining intensity was scored 0 to 4 (0, absent; 1, weak; 2, intermediate; 3, strong; or 4, very strong). The percentage of positively stained cells was scored 0 to 4 (0, absent; 1, 1–<10%; 2, 10%–<50%; 3, 50%–<75%; or 4, ≥75%). Both scores were multiplied to obtain an immunoreactivity score. Statistical analysis was performed in statistical software package R using the paired Wilcoxon test and Spearman correlation.

## Results

### RIOK1 Is Significantly Up-Regulated in PCa

**Figure 1:**
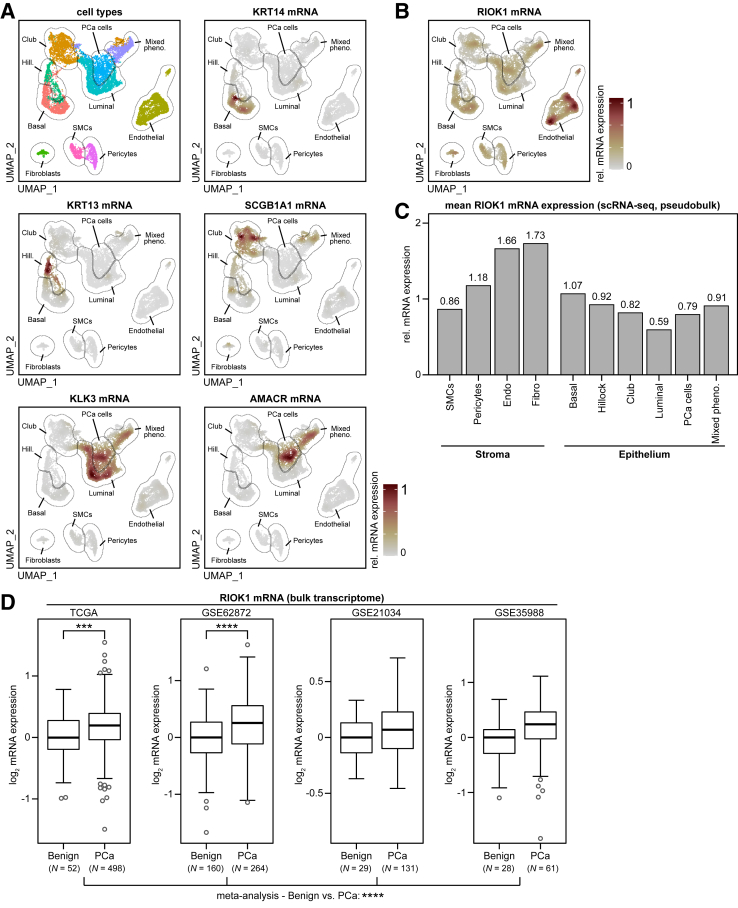
RIOK1 is overexpressed in PCa at the mRNA level. **A** and **B:** Re-analysis of the single-cell RNA-seq GSE193337 (*https://www.ncbi.nlm.nih.gov/geo*; accession number GSE193337) containing four PCa and adjacent benign tissue samples showing the clustering/cell type marker gene expression (**A**) and log_2_ RIOK1 mRNA expression as shown in a two-dimensional uniform manifold approximation and projection (UMAP) plot (**B**). **C:** Bar graph, after pseudobulk summarization, of RIOK1 mRNA expression (linear scale) across different cell types. **D:** RIOK1 bulk mRNA expression and meta-analysis–based differential gene expression statistics in PCa and benign prostate tissue of four independent public transcriptome data sets from The Cancer Genome Atlas (TCGA) (*https://portal.gdc.cancer.gov/projects/TCGA-PRAD*, last accessed May 30, 2023; accession TCGA-PRAD), and Gene Expression Omnibus (*https://www.ncbi.nlm.nih.gov/geo*; accession numbers GSE62872, GSE21034, and GSE35988). Data are expressed as means (**C**) or as medians (interquartile range) [minimum, maximum] and outliers (**D**). ∗∗∗*P* < 0.001, ∗∗∗∗*P* < 0.0001. AMACR, α-methylacyl–coenzyme A racemase; Hill., Hillock; KLK, kallikrein; KRT, keratin; SCGB1A1, secretoglobin family 1A member 1; scRNA, small conditional RNA; SMC, structural maintenance of chromosomes protein.

To assess the expression of RIOK1 in PCa, data from a publicly available single-cell RNA-seq data set, consisting of benign and cancerous tissue samples from four patients who underwent radical prostatectomy, were reanalyzed.^22^ Cell-type annotation was performed using well-known markers (Figure 1A and Supplemental Figure S1). While a high concordance between the cell-type markers and individual clusters was observed, one cluster (termed mixed pheno.) was composed of various cell types. RIOK1 was expressed in all cell types (Figure 1, B and C). Both techniques showed above-median RIOK1 expression in endothelial cells. The results on fibroblasts were not completely consistent due to technical challenges caused by the low cell number in this cluster. The expression of RIOK1 mRNA was greater in the stromal compartment (smooth muscle cells, pericytes, endothelial cells, fibroblasts) compared to that in the epithelial compartment (basal cells, hillock cells, club cells, luminal cells, PCa cells, mixed phenotype cells) (adjusted *P* = 6.9 × 10^−55^). In addition, the increase in RIOK1 mRNA expression was small but significant in PCa cells compared to that in luminal cells (difference, +33.9%; adjusted *P* = 6.1 × 10^−7^).

Next, a meta-analysis of data from four publicly available bulk transcriptome data sets (representing 269 benign prostate and 954 PCa tissue samples) was performed to validate the increase of RIOK1 mRNA expression in PCa cells. Combined analysis of these data sets confirmed a small but significant increase in RIOK1 mRNA expression in PCa tissue compared to benign tissue (median increases, 14%, 19%, 5% and 18%, respectively; combined adjusted *P* = 7.0 × 10^−5^) (Figure 1D).

**Figure 2:**
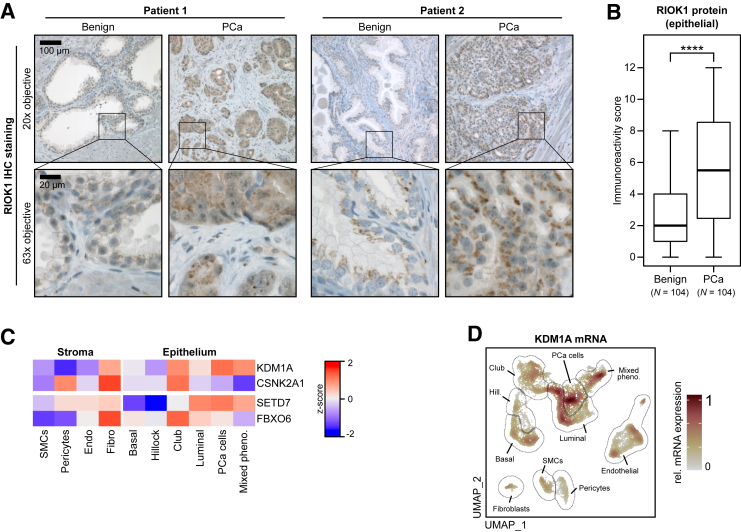
RIOK1 is strongly overexpressed in PCa at the protein level. **A:** IHC RIOK1 staining of PCa and adjacent benign prostate tissue from two patients. Boxed regions in **upper panels** correspond to **lower panels** at higher magnification. **B:** Quantification of IHC analysis RIOK1 staining (immunoreactivity score) in epithelial cells in the tissue microarray. **C:** Heatmap showing the expression (*z*-score–normalized pseudobulk) of four post-translational RIOK1 regulators across different cell types in the re-analyzed single-cell RNA-seq data set (*https://www.ncbi.nlm.nih.gov/geo*; accession number GSE193337). **D:** Log_2_ mRNA expression of the post-translational RIOK1 regulator lysine-specific histone demethylase (KDM)-1A. Data are expressed as medians (interquartile ranges) [minimum, maximum]. ∗∗∗∗*P* < 0.0001. Scale bars: 100 μm (original magnification, ×20; **A**, **upper panel**); 20 μm (original magnification, ×63; **A**, **lower panel**). CSNK2, casein kinase II; FBXO, F-box only protein; SETD7, histone-lysine *N*-methyltransferase SETD7; SMC, structural maintenance of chromosomes protein; UMAP, uniform manifold approximation and projection.

Immunohistochemistry (IHC) analysis was performed with an anti-RIOK1 antibody to measure RIOK1 levels in tissue samples from PCa patients who had undergone radical prostatectomy. Specificity of the RIOK1 IHC staining was confirmed with RIOK1 overexpression/knockdown cell culture samples and with an isotype control (Supplemental Figure S2, A–C). In contrast to the single-cell RNA-seq results, RIOK1 staining intensity was very low in the stromal compartment and much greater in the epithelial compartment (Figure 2A). However, in line with the RNA results, a much stronger staining was observed in PCa tissue samples. Therefore, the epithelial RIOK1 expression was quantified in a tissue microarray containing cancerous and adjacent benign tissue samples from 120 PCa patients (Table 1). A total of 16 samples were excluded from the analysis due to missing cores, leaving 104 paired benign/PCa samples. The epithelial RIOK1 protein level was significantly elevated (by threefold) in PCa samples compared to benign samples (Figure 2B). However, there were no correlations between RIOK1 and Gleason score/International Society of Urologic Pathologists grade (Supplemental Figure S2D), tumor stage (Supplemental Figure S2E), or survival (Supplemental Figure S2F).

**Table 1:** Patient Characteristics of the IBK1 Tissue Microarray

Characteristic	Value
Age at diagnosis, mean (range), years	61.5 (41.3–77.9)
PSA at diagnosis, mean (range), ng/mL	5.67 (1.75–32)
ISUP grade, *n* (%)	
1 (GS ≤6)	38 (31.7)
2 (GS 3 + 4)	39 (32.5)
3 (GS 4 + 3)	17 (14.2)
4 (GS 8)	7 (5.8)
5 (GS ≥9)	19 (15.8)
T stage, *n* (%)	
pT2	75 (62.5)
pT3	43 (35.8)
pT4	2 (1.7)
Biochemical relapse, *n* (%)	58 (48.33)
Years to relapse, mean (range)	2.81 (0.14–10.58)
Years of follow-up, mean (range)	9.38 (3.27–20.61)

GS, Gleason score; IBK, Innsbruck; ISUP, International Society of Urologic Pathologists; PSA, prostate-specific antigen.

RIOK1 protein expression is regulated by post-translational mechanisms.^10^ Specifically, RIOK1 methylation by histone-lysine *N*-methyltransferase SETD7 leads to ubiquitination by the F-box only protein (FBXO)-6–containing E3 ubiquitin ligase S-phase kinase-associated protein–cullin 1-F-box (SCF) complex, whereas lysine-specific histone demethylase (KDM)-1A and casein kinase II (CSNK2)-α_1_ block this process. Therefore, whether the expression of these genes might explain the differences between RIOK1 transcript and protein levels was explored. Differences in the expression of all four genes were observed across the different cell types in the single-cell RNA-seq data set (Figure 2C). Of note, mRNA expression of KDM1A (Figure 2D) was significantly elevated in the epithelial compartment (adjusted *P* = 6.5 × 10^−92^) as well as in PCa cells (adjusted *P* = 1.4 × 10^−18^).

Taken together, the results from the present study demonstrate that RIOK1 is up-regulated in PCa tissue, likely due to a combination of transcriptional and post-translational mechanisms.

### RIOK1 Is a Downstream Target of MYC

To study the functional and regulatory landscape of RIOK1, a correlation analysis was performed on data from tissue samples of primary PCa (TCGA data set, *n* = 498) and castration-resistant PCa (SU2C data set, *n* = 259) from two publicly available RNA-seq data sets.^9^^,^^23^ On pathway analysis (Table 2 and Supplemental Table S1), RIOK1 mRNA level was positively correlated with several proliferative (E2F_TARGETS, G2M_CHECKPOINT, MYC_TARGETS_V1/2) and protein synthesis/degradation related pathways (UPR, MTORC1_SIGNALING). Whereas in previous reports RIOK1 expression was correlated with hormone receptor status and phosphatidylinositol 3-kinase (PI3K)/AKT signaling in breast cancer and glioma,^11^^,^^13^ respectively, no consistent association of RIOK1 mRNA expression with AR and PI3K/AKT target gene sets in PCa was observed.

**Table 2:** RIOK1 Co-Expression Pathway Analysis of the MSigDB Hallmark Pathways in Early (TCGA^9^) and Castration Resistant (SU2C^23^) PCa RNA-Seq Data Sets

Correlation	Adjusted *P*	
Positive correlation with RIOK1		
MSigDB HALLMARK pathway	TCGA	SU2C
E2F_TARGETS	4.9 × 10^−21^	4.4 × 10^−26^
G2M_CHECKPOINT	8.5 × 10^−21^	4.0 × 10^−16^
MYC_TARGETS_V1	2.2 × 10^−14^	6.1 × 10^−36^
MYC_TARGETS_V2	2.2 × 10^−06^	1.3 × 10^−11^
UNFOLDED_PROTEIN_RESPONSE	1.1 × 10^−04^	2.1 × 10^−04^
MTORC1_SIGNALING	5.1 × 10^−03^	4.1 × 10^−04^
Negative correlation with RIOK1		
MSigDB HALLMARK pathway	TCGA	SU2C
MYOGENESIS	1.4 × 10^−07^	1.0 × 10^−04^
KRAS_SIGNALING_DN	1.4 × 10^−02^	8.0 × 10^−04^
XENOBIOTIC_METABOLISM	1.7 × 10^−02^	2.1 × 10^−04^
ESTROGEN_RESPONSE_LATE	3.4 × 10^−02^	7.4 × 10^−03^
COAGULATION	4.9 × 10^−02^	6.0 × 10^−08^

SU2C, Stand Up To Cancer; TCGA, The Cancer Genome Atlas.

**Figure 3:**
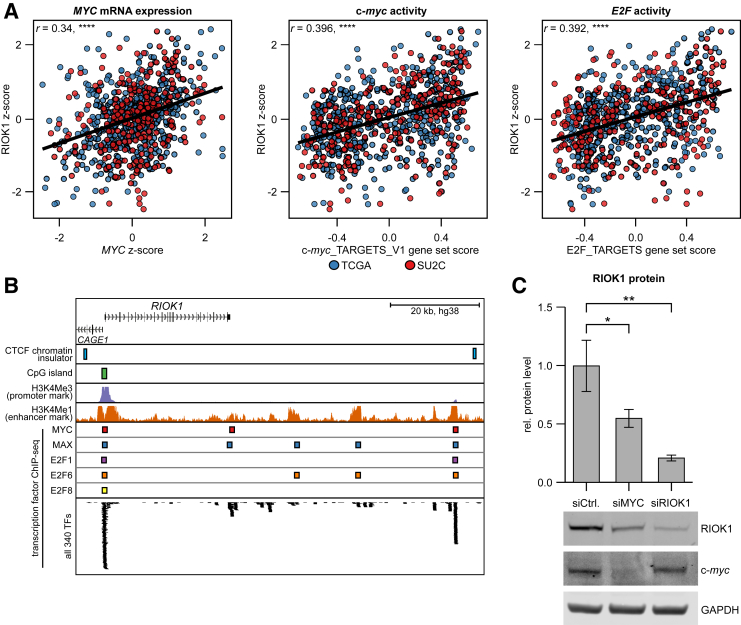
RIOK1 is downstream target of the c-*myc*/E2F transcription factor axis. **A:** Correlation of RIOK1 mRNA expression (*z*-score) with c-*myc* mRNA expression and *MYC/E2F* target gene set activity (GSVA software version 1.44.4). **B:** Histone marks and transcription factor chromatin immunoprecipitation sequencing peaks in the genomic region surrounding RIOK1 from public data sets available via the University of California–Santa Cruz genome browser and ENCODE project (*https://genome.ucsc.edu/ENCODE*, last accessed May 30, 2023). **C:** Detection of RIOK1 protein expression by Western blot analysis upon siRNA-mediated knockdown of c-*myc* in PC3 cells at 72 hours after transfection. Data are expressed as means (95% CI). ∗*P* < 0.05, ∗∗*P* < 0.01, and ∗∗∗∗*P* < 0.0001. ChIP, chromatin immunoprecipitation; CTCF, transcriptional repressor CTCF; GAPDH, glyceraldehyde phosphate dehydrogenase; MAX, protein max; TFs, transcription factors.

RIOK1 was previously indicated as a direct c-*myc* downstream target in a lung cancer cell line,^26^ which might explain the present pathway-analysis results. Therefore, the impact of c-*myc* and the c-*myc–*regulated E2F family of cell-cycle master regulators on RIOK1 expression was investigated. Transcription factor activity scores for c-*myc* and E2F were calculated using GSVA and the well-established MSigDB target gene set signatures.^27^ RIOK1 mRNA expression was significantly correlated with c-*myc* mRNA expression and activity of the c-*myc*/E2F transcription factor axis (Figure 3A). Next, publicly available transcription factor chromatin immunoprecipitation sequencing data from the ENCODE project^25^ were investigated to confirm direct binding of c-*myc* and E2F type transcription factors in the vicinity of RIOK1 in a wide panel of cell lines. c-*myc* [and its heterodimerization partner, protein max (MAX)], as well as several E2F transcription factors (ie, E2F1), bound to the promoter and various enhancer regions of *RIOK1* (Figure 3B). Of note, a similar regulation pattern of the RIOK1 protein stability regulator *KDM1A* was observed (Supplemental Figure S3, A and B), suggesting co-regulation of the two genes. Subsequently, siRNA-based RNA interference experiments were performed to confirm that c-*myc* is a regulator of RIOK1 expression. RIOK1 protein expression was significantly reduced, by nearly 50%, with c-*myc* knockdown (Figure 3C). In contrast, RIOK1 knockdown did not have any short-term (72 hours) effects on c-*myc* protein expression (Supplemental Figure S3C).

In summary, the results in the previous paragraph indicate that RIOK1 is a downstream target of the cell-cycle master regulator c-*myc*/E2F transcription factor axis.

### RIOK1 Is Essential for PCa Cells

**Figure 4:**
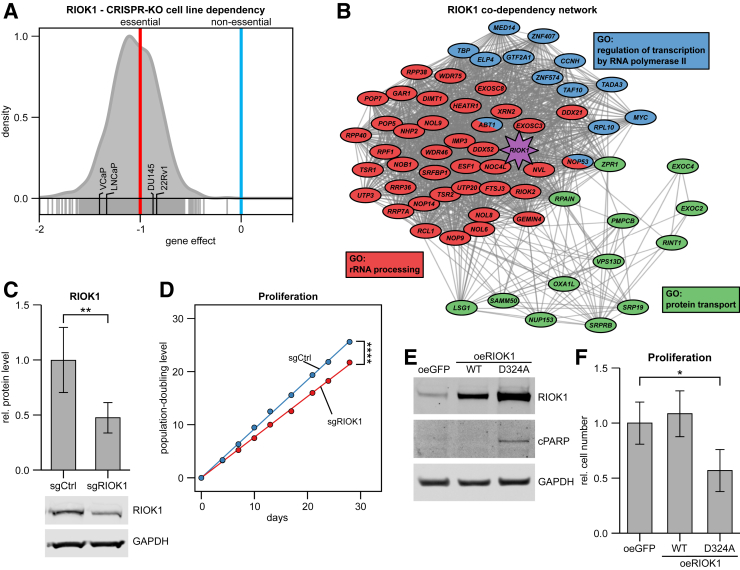
*RIOK1* is an essential gene in PCa. **A:***RIOK1* gene dependency in 1086 cell lines of a public clustered regularly interspaced short palindromic repeats (CRISPR)-knockout (KO) data set. **B:** Correlation of RIOK1 gene dependency against all other genes summarized as co-dependency network of the top three pathways. **C** and **D:** RIOK1 protein expression (**C**) and population growth curves (**D**) upon CRISPR interference–based RIOK1 knockdown in PC3 cells. **E:** Exemplary Western blot showing RIOK1 and cleaved PARP protein expression upon overexpression (oe) of wild type and dominant-negative (D324A mutant) RIOK1 in PC3 cells. **F:** Proliferation of PC3 cells upon overexpression of wild type and dominant-negative (D324A mutant) RIOK1 after 72 hours after transfection. Data are expressed as means (95% CI). ∗*P* < 0.05, ∗∗*P* < 0.01, and ∗∗∗∗*P* < 0.0001. GAPDH, glyceraldehyde phosphate dehydrogenase; GFP, green fluorescent protein; sg, single-guide; WT, wild-type.

Next, the genome-wide loss-of-function data from the DepMap project^24^ were used to investigate the vulnerability of cancer cells to CRISPR/CRISPR-associated endonuclease (CAS)-9–mediated *RIOK1* knockout. RIOK1 was found to be essential in 1080 of 1086 tested cancer cell lines (Figure 4A), including the PCa cell lines VCaP, LNCaP, DU145, and 22Rv1. To gain further information about the network of *RIOK1* co-dependencies, correlation analysis between *RIOK1* and all other genes in the CRISPR data set was performed. A significant positive correlation in dependency between *RIOK1* and 143 essential genes was observed. On pathway analysis (Figure 4B), a strong co-dependency of *RIOK1* with genes involved in rRNA processing, protein transport, and regulation of transcription was found. Of note, a significant positive co-dependency between *RIOK1* and *MYC* was found (ρ = 0.13, adjusted *P* = 0.002).

To corroborate these observations, RIOK1 knockdown experiments were performed using CRISPR interference. Specifically, PC3 cells stably expressing a catalytically dead (d)-CAS9 protein, fused to a transcriptional repressor domain (*ZIM3-KRAB-dCAS9*) and a sgRNA targeting the promoter region of *RIOK1* (sgRIOK1), were generated. RIOK1 expression was reduced by 74% on mRNA (quantitative RT-PCR) and by 53% at the protein level (anti-RIOK1 Western blot) (Figure 4C and Supplemental Figure S4). To evaluate the long-term consequence of RIOK1 knockdown, the growth of these cells was measured over 4 weeks, with a significant reduction in the growth rate of RIOK1-depleted cells (Figure 4D). Subsequently, overexpression experiments were performed by transient transfection of PC3 cells with plasmids encoding for wild-type RIOK1 and the kinase/ATPase-dead RIOK1-D324A mutant (Figure 4E). Overexpression of wild-type RIOK1 (oeRIOK1-WT) had no effect on proliferation after 72 hours, whereas with overexpression of the catalytically RIOK1 mutant (oeRIOK1-D324A), cell proliferation was significantly reduced, by nearly 50% (Figure 4F). In addition, an induction of the apoptosis marker cPARP was observed (Figure 4E).

Taken together, the present findings demonstrate that the expression of functionally active RIOK1 is essential for PCa cell proliferation, indicating that RIOK1 could be a valid therapeutic target in the treatment of patients with PCa.

### Toyocamycin Is an Efficient Inhibitor of RIOK1 and Induces Apoptosis

**Figure 5:**
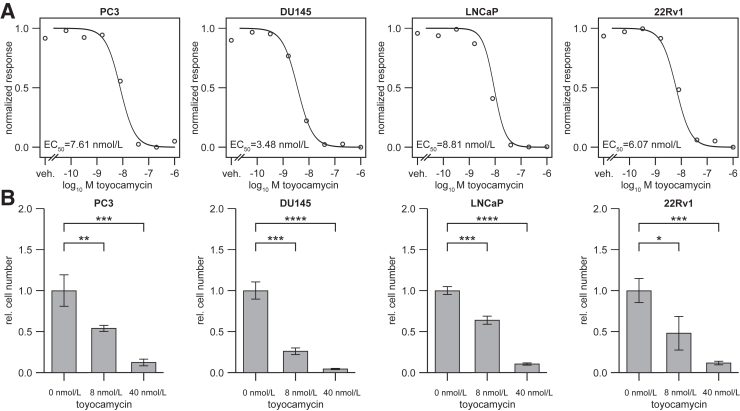
Toyocamycin is a potent inhibitor of PCa cell growth. **A:** Dose-response curves after treatment of various PCa cell lines with the RIOK1 inhibitor toyocamycin after 72 hours. **B:** Bar plots showing the fold-changes at selected toyocamycin concentrations at 72 hours after treatment. Data are expressed as means (95% CI). ∗*P* < 0.05, ∗∗*P* < 0.01, ∗∗∗*P* < 0.001, and ∗∗∗∗*P* < 0.0001.

Next, the therapeutic potential of toyocamycin, a small-molecule inhibitor of RIOK1, was tested. Toyocamycin binds to the ATP binding pocket of RIOK1, which inhibits the phosphorylation activity of RIOK1, similar to the RIOK1-D324A mutant.^28^^,^^29^ In dose-response experiments in AR-negative (PC3, DU145) and AR-positive (LNCaP, 22Rv1) PCa cell lines, PCa cell growth was highly inhibited with toyocamycin with EC_50_ values ranging from 3.5 to 8.8 nmol/L (Figure 5, A and B).

**Figure 6:**
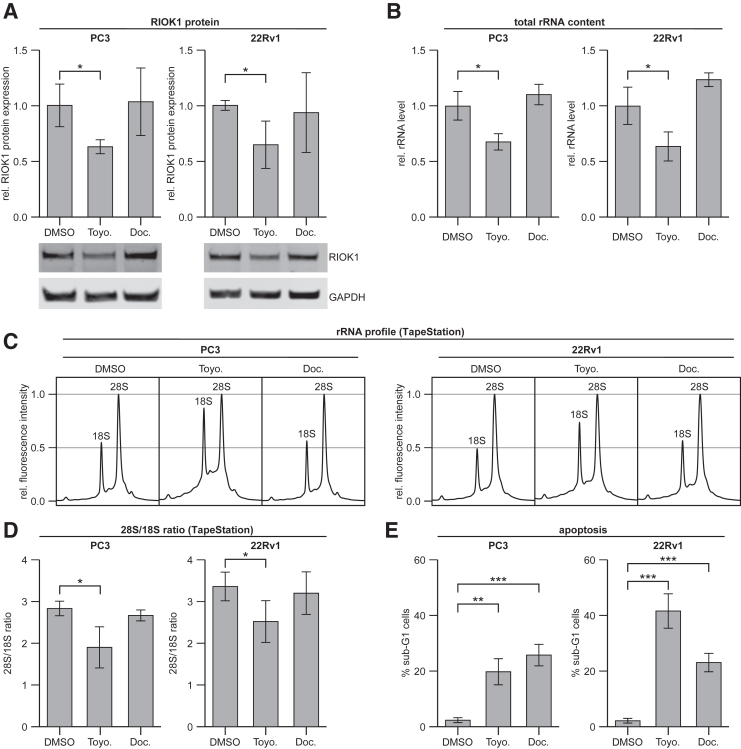
Toyocamycin reduces rRNA levels and induces apoptosis in PCa cell lines. **A–D:** RIOK1 protein expression (**A**), total rRNA content per cell (**B**), rRNA profile (**C**), and quantification (**D**) of the 28S/18S rRNA ratio at 24 hours after treatment with 100 nmol/L toyocamycin (Toyo.) or docetaxel (Doc.). **E:** Percentage sub-G_1_ cells after 72 hours of 100 nmol/L toyocamycin or docetaxel treatment. Data are expressed as means (95% CI). ∗*P* < 0.05, ∗∗*P* < 0.01, and ∗∗∗*P* < 0.001. DMSO, dimethyl sulfoxide; GAPDH, glyceraldehyde phosphate dehydrogenase.

To investigate the underlying mechanism of toyocamycin-related toxicity in PCa cells, short-term experiments (24 hours) in PC3 and 22Rv1 cells at a concentration of 100 nmol/L were performed. To differentiate between toyocamycin-specific and general cell death related alterations, the clinically used chemotherapeutic drug docetaxel (100 nmol/L) was included in these experiments. RIOK1 protein expression was significantly reduced within 24 hours after biochemical inhibition of RIOK1 (Figure 6A), which might indicate that RIOK1 auto-activates its own expression, as observed in budding yeast.^30^ Next, the impact of toyocamycin on rRNA content was measured, given the importance of RIOK1 in the production of rRNA and in the processing of the 18S-E pre-rRNA.^6^^,^^31^ Total rRNA content per cell was significantly reduced, by >30%, in both cell lines (Figure 6B). Analysis of the rRNA profile revealed a significant shift in the ratio between 18S and 28S rRNA, with a relative accumulation of 18S rRNA (Figure 6, C and D). In contrast, RIOK1 protein expression, rRNA content per cell, and the rRNA profile were not significantly affected with docetaxel.

Lastly, to assess whether toyocamycin induces apoptosis in PCa cells, the sub-G_1_ fraction was measured in PC3 and 22Rv1 cells using fluorescence-activated cell sorting analysis after 72 hours of toyocamycin treatment, and the effect was compared to that with docetaxel treatment. With the two drugs at a concentration of 100 nmol/L, apoptosis was significantly induced at 72 hours after treatment (Figure 6E). Of note, the percentage of sub-G_1_ cells with toyocamycin treatment was similar to or greater than that with the well-established chemotherapeutic drug docetaxel.

Taken together, these results suggest that biochemical inhibition of RIOK1 with toyocamycin is a potent inducer of programed cell death in PCa cells.

## Discussion

The findings from the present study demonstrate that the highly conserved atypical protein kinase/ATPase RIOK1 is elevated in PCa and essential for the proliferation/survival of PCa cells. The finding that RIOK1 was up-regulated at the protein level in PCa cells extends the number of cancer types with proved RIOK1 overexpression.^10^, ^11^, ^12^^,^^14^^,^^15^ In a study by Huang et al^13^ in breast cancer, proliferation was inhibited and apoptosis was induced with knockdown of RIOK1, consistent with the present results in PCa after RIOK1 inhibition. Huang et al^13^ also demonstrated that elevated RIOK1 expression was associated with higher tumor grade and was correlated with absent hormone receptor expression.^13^ In contrast, in the present study, no correlations were found between RIOK1 mRNA expression and clinical parameters such as tumor stage, International Society of Urologic Pathologists grade/Gleason score, and biochemical relapse-free survival. Furthermore, no association was found between RIOK1 mRNA expression and AR target gene activity in PCa. An oncogenic role of *RIOK1* in glioma has been suggested, in which RIOK1 is co-expressed with AKT.^11^ However, in PCa tissue, no major association between RIOK1 mRNA expression and PI3K/AKT gene set activity was found. In lung cancer, RIOK1 is reported to have maintained cell survival and a diminished therapeutic effect of cisplatin, with lower survival in patients with greater RIOK1 expression compared to that in their counterparts.^12^ Another example of a tumorigenic role of RIOK1 is colorectal cancer, in which RIOK1 causes p53 degradation and resistance to radiation therapy.^15^

Of note, the increase in RIOK1 protein expression in PCa tissue was threefold, which is much greater than the 5% to 30% increase observed at the mRNA level. This finding suggests a strong post-translational regulation of RIOK1. Stability of the RIOK1 protein level is heavily regulated by SETD7-dependent RIOK1-K411 methylation, which leads to ubiquitination via FBXO6 and proteasomal degradation. In contrast, RIOK1 stability is increased on phosphorylation by the CSNK2A1 complex and KDM1A-mediated RIOK1 demethylation.^10^ Variable mRNA expression of all four RIOK1 post-translational regulators was observed across prostate cell types. In particular, KDM1A mRNA expression was significantly elevated in PCa cells and within the epithelial compartment in general. In experiments in colorectal cancer tissue samples, RIOK1 phosphorylation and protein expression of KDM1A/CSNK2A1 are positively correlated with RIOK1 protein expression, whereas RIOK1 methylation is negatively correlated with protein expression of SETD7/FBXO6.^10^ Based on the results from the present study, it is clear that further studies are necessary to evaluate the post-translational regulation of RIOK1 in PCa, which is of particular interest given that KDM1A activates a PCa gene network associated with aggressiveness, thus promoting castration resistance.^32^ *KDM1A* is important for the development of neuroendocrine PCa through splicing and regulation of neuroendocrine genes.^33^ Thus, it may be important to further study the RIOK1/KDM1A relationship in neuroendocrine PCa, which cannot be targeted by hormone therapies.

The results from several laboratories^13^, ^14^, ^15^ have implied that *RIOK1* may affect tumor growth in multiple ways. That is consistent with the present finding that RIOK1 is a downstream target of the c-*myc*/E2F cell-cycle master regulators. *MYC* is a key oncogene in PCa, and high expression of c-*myc* in PCa has been associated with reduced overall survival and poor prognosis.^34^ Amplification of *MYC* in castration-resistant PCa has been reported,^35^ and hyperactivation of the c-*myc*/E2F transcription factor axis has been observed in AR-indifferent, anti-androgen–resistant PCa cells.^36^ RIOK1 has an essential function as a ribosomal biogenesis factor in the final steps of pre-40S ribosomal maturation.^6^ Therefore, RIOK1 is likely an important factor in the well-recognized function of c-*myc* as a master regulator of ribosome biogenesis.^37^ Expression of c-*myc* is a determinant of activity of the BET bromodomain–containing protein (BRD)-4, which regulates AR transcriptional activity.^38^ Future studies could investigate whether *RIOK1*, as a c-*myc* target gene, has a direct or indirect role in the regulation of androgen responsiveness in PCa. RIOK1 expression in PCa is also regulated by E2F transcription factors, which are main proliferative regulators in PCa. E2F family members are known to contribute to prostate tumorigenesis by up-regulation of serum and glucocorticoid-induced protein kinase 1.^39^ Glucocorticoid receptors are expressed in metastatic PCa that is resistant to endocrine therapy and chemotherapy.^40^ Taken together, the findings from recent publications and the present study show that the c-*myc*/E2F transcription factor axis may promote prostate carcinogenesis by multiple mechanisms, including up-regulation of RIOK1.

The present results also show a co-dependency of *RIOK1* and genes involved in rRNA processing, protein transport, and regulation of transcription, making *RIOK1* an interesting target in the development of therapies for PCa. *RIOK1* is of particular interest, given that translation initiation in PCa is regulated by the eukaryotic translation initiation factor (eIF)-4E cap-binding protein, which is highly phosphorylated in therapy-resistant PCa.^41^ Due to the importance of RIOK1 in ribosome biogenesis, it is not surprising that RIOK1 inhibition caused massive effects in androgen-sensitive and -insensitive cell lines. These findings suggest that RIOK1 inhibition may have broader potential in the treatment of patients with PCa. In colon cancer, the therapeutic potential of toyocamycin has been demonstrated *in vitro* and *in vivo*.^15^ Inhibition of growth in LNCaP cells by toyocamycin was reported by Park et al,^42^ who observed that toyocamycin caused a G_1_ phase arrest and up-regulation of the inhibitor p21. The effects of toyocamycin in PC3 cells could be attributable to the induction of apoptosis and mitochondrial dysfunction.^43^ In those experiments, toyocamycin was not studied in relation to RIOK1. Direct inhibition of the RIOK1/ATPase domain by toyocamycin has been confirmed with thermal shift assays and by X-ray crystallography,^28^ but toyocamycin has been shown to interact also with other targets, such as cyclin-dependent kinase (CDK)-9 and X-box–binding protein (XBP)-1 splicing.^44^^,^^45^ To ensure that the observed effects are due to RIOK1 inhibition, the effects on proliferation and apoptosis were confirmed by overexpression of a catalytically dead RIOK1 mutant (mutation of the central D324A position in the kinase/ATPase center). Furthermore, the findings from the present experiments suggest that toyocamycin led to a reduction in RIOK1 protein expression/rRNA content and a shift in the 28S/18S ratio toward 18S rRNA. In previous studies of human RIOK1 and the budding yeast ortholog (Rio1), RIOK1 depletion blocked maturation of 18S rRNA and led to the accumulation of 18S-E pre-rRNA.^29^^,^^46^ Of note, overexpression of kinase/ATPase-dead Rio1 was reported to lead to a similar accumulation of pre-rRNAs.^31^ The technique used in this study cannot be used for differentiating between 18S-E pre-rRNA and 18S rRNA; thus, further studies might be required to validate this finding in PCa cells. Taken together, these findings demonstrate that biochemical (toyocamycin) and genetic (D324A mutant) blockades of the *RIOK1* kinase/ATPase domain have severe effects on rRNA biogenesis, proliferation, and apoptosis. However, further studies of potential RIOK1-independent effects of toyocamycin are required.

It is interesting to note that the overexpression of the RIOK1-D324A mutant, similar to that of toyocamycin, effectively inhibited cell proliferation and apoptosis. In contrast, the CRISPR interference-based knockdown of RIOK1 had only mild effects on proliferation and did not induce apoptosis. Given that the reduction in RIOK1 protein level was only 53%, it is possible that sufficient RIOK1 remains to prevent apoptosis induction. However, it is important to note that previous studies have shown that kinase/ATPase-deficient yeast Rio1 mutants are stuck on pre-40S particles, which traps other rRNA biogenesis factors and leads to translation-initiation defects.^29^^,^^31^ Therefore, inhibition of the *RIOK1* kinase/ATPase domain is more likely to cause a different phenotype than a reduction in RIOK1 expression. However, further studies are required to determine whether antineoplastic effects are superior with the inhibition of the *RIOK1* kinase/ATPase domain compared with RIOK1 down-regulation. Of note, a recent drug-repurposing study predicted the drug levosimendan to bind to the RIOK1 ATP-binding pocket and to act as a potential RIOK1 inhibitor.^47^ The antineoplastic efficacy of levosimendan has been tested in 200 cancer cell lines, and hematopoietic lymphoma cell lines were particularly sensitive. Therefore, at least two chemically distinct lead compounds are available for the development of further RIOK1 inhibitors.

Taken together, the findings from the present study demonstrate that the atypical kinase/ATPase RIOK1 is up-regulated in PCa tissue specimens and that it is an essential component of several important oncogenic processes. It is a part of the *MYC* oncogene network and as such it could be considered a valid therapeutic target for future strategies of eradicating PCa.

## Supplemental Data

Supplemental Figure S1 Re-analysis of the single-cell RNA-seq GSE193337 (*https://www.ncbi.nlm.nih.gov/geo*; accession number GSE193337) containing four samples of PCa and adjacent benign tissue showing the cell type marker gene expression of the stromal clusters.

Supplemental Figure S2 **A** and **B:** IHC RIOK1 staining (**A**) and confirmatory Western blot (**B**) of a PCa cell line with unaltered RIOK1 expression, upon overexpression of RIOK1 and upon siRNA-mediated RIOK1 knockdown. **C:** IHC staining of prostate tissue with an isotype control and the anti-RIOK1 antibody. **D** and **E:** Epithelial RIOK1 staining intensity of the PCa tissue samples in the tissue microarray (TMA) split by International Society of Urologic Pathologists (ISUP) grade (**D**) and tumor stage (**E**). **F:** Kaplan-Meier plot showing biochemical relapse-free survival of patients with below-median (*n* = 54) and above-median (*n* = 53) RIOK1 protein expression. Scale bars: 100 μm (original magnification, ×20; **A** and **C**, **upper panel****s**); 20 μm (original magnification, ×63; **A** and **C**, **lower panel****s**).

Supplemental Figure S3 **A:** Correlation of lysine-specific histone demethylase (KDM)-1A mRNA expression (*z*-score) with c-*myc* mRNA expression and *MYC/E2F* target gene set activity (GSVA software version 1.44.4). **B:** Histone marks and transcription factor chromatin immunoprecipitation sequencing peaks in the genomic region surrounding KDM1A from public data sets available via the University of California–Santa Cruz genome browser and the ENCODE project (https://genome.ucsc.edu/ENCODE, last accessed May 30, 2023). **C:** Detection of c-*myc* protein expression by Western blot analysis upon siRNA-mediated knockdown of RIOK1 in PC3 cells. Data are expressed as means (95% CI). ∗∗*P* < 0.01, ∗∗∗∗*P* < 0.0001.

Supplemental Figure S4 RIOK1 mRNA expression upon CRISPR interference–based RIOK1 knockdown in PC3 cells. Data are expressed as means (95% CI). ∗∗∗∗*P* < 0.0001.
