# Supplementary figures and images for: The Oncogenic Protein Kinase/ATPase RIOK1 Is Up-Regulated via the c-myc/E2F Transcription Factor Axis in Prostate Cancer
Source: Am J Pathol. 2023 Jun 9;193(9):1284–97. doi: 10.1016/j.ajpath.2023.05.013 (PMC12178385; doi:10.1016/j.ajpath.2023.05.013)

# Supplementary Figure 1

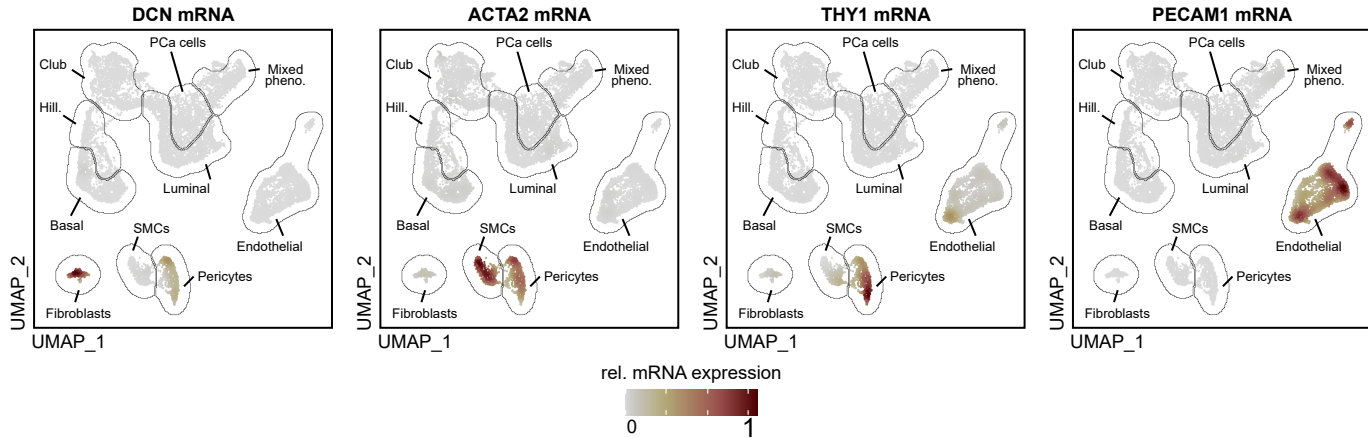

Supplement: Supplemental Figure S1 — Re-analysis of the single-cell RNA-seq GSE193337 (https://www.ncbi.nlm.nih.gov/geo; accession number GSE193337) containing four samples of PCa and adjacent benign tissue showing the cell type marker gene expression of the stromal clusters. [file mmc1.pdf]

# Supplementary Figure 2

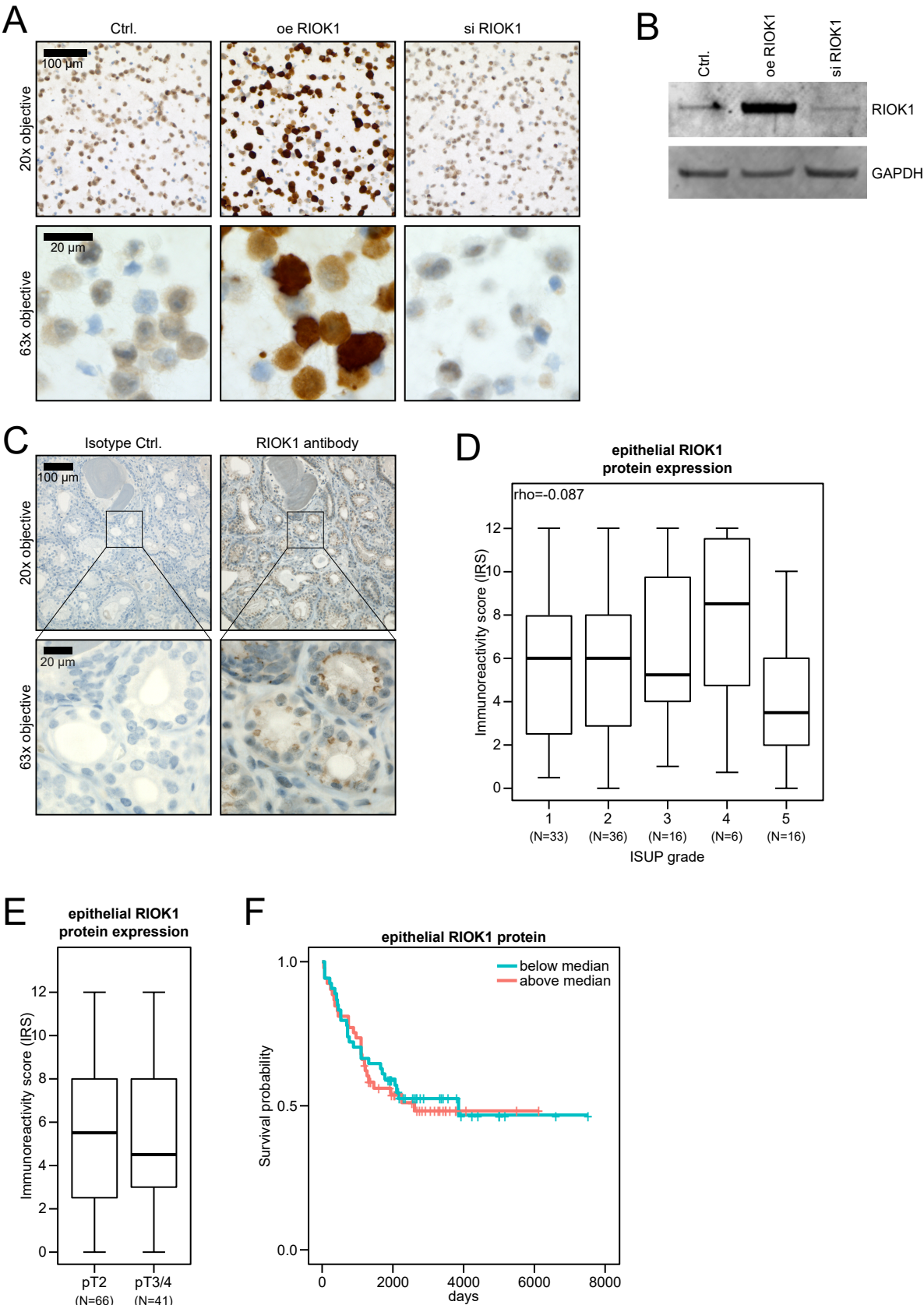

Supplement: Supplemental Figure S2 — A and B: IHC RIOK1 staining (A) and confirmatory Western blot (B) of a PCa cell line with unaltered RIOK1 expression, upon overexpression of RIOK1 and upon siRNA-mediated RIOK1 knockdown. C: IHC staining of prostate tissue with an isotype control and the anti-RIOK1 antibody. D and E: Epithelial RIOK1 staining intensity of the PCa tissue samples in the tissue microarray (TMA) split by International Society of Urologic Pathologists (ISUP) grade (D) and tumor stage (E). F: Kaplan-Meier plot showing biochemical relapse-free survival of patients with below-median (n = 54) and above-median (n = 53) RIOK1 protein expression. Scale bars: 100 μm (original magnification, ×20; A and C, upper panels); 20 μm (original magnification, ×63; A and C, lower panels). [file mmc2.pdf]

# Supplementary Figure 3

A

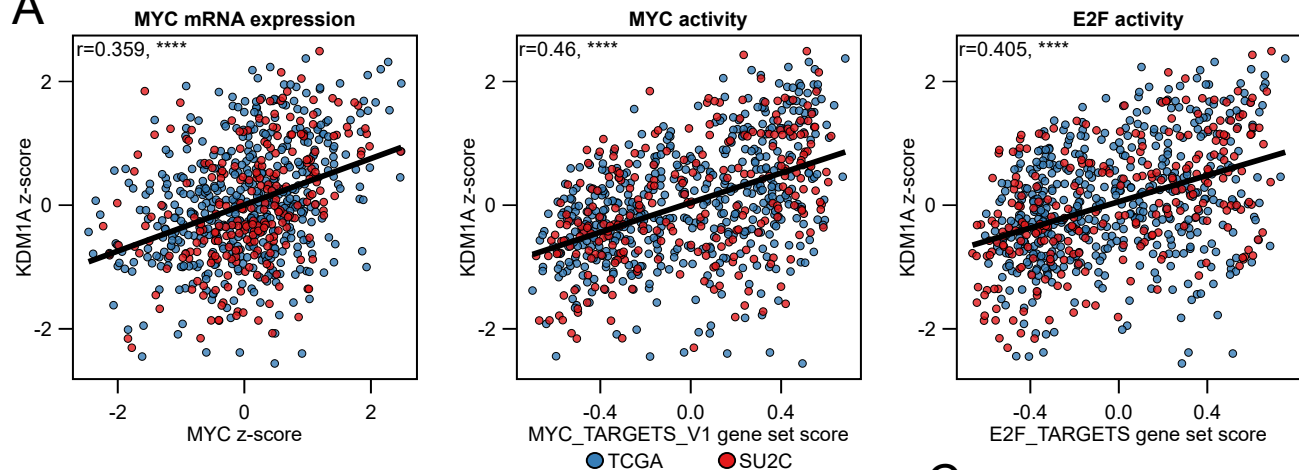

B

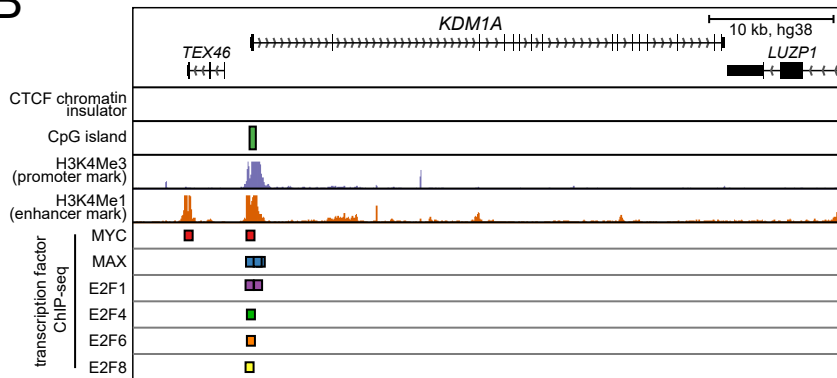

C

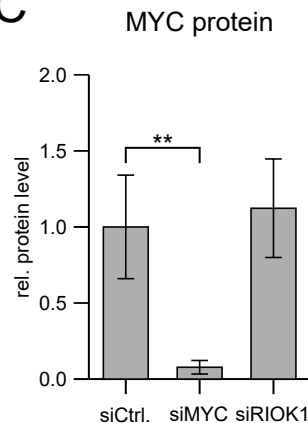

Supplement: Supplemental Figure S3 — A: Correlation of lysine-specific histone demethylase (KDM)-1A mRNA expression (z-score) with c-myc mRNA expression and MYC/E2F target gene set activity (GSVA software version 1.44.4). B: Histone marks and transcription factor chromatin immunoprecipitation sequencing peaks in the genomic region surrounding KDM1A from public data sets available via the University of California–Santa Cruz genome browser and the ENCODE project (https://genome.ucsc.edu/ENCODE, last accessed May 30, 2023). C: Detection of c-myc protein expression by Western blot analysis upon siRNA-mediated knockdown of RIOK1 in PC3 cells. Data are expressed as means (95% CI). ∗∗P < 0.01, ∗∗∗∗P < 0.0001. [file mmc3.pdf]

# Supplementary Figure 4

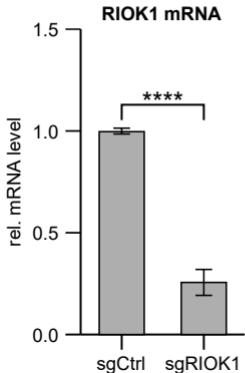

Supplement: Supplemental Figure S4 — RIOK1 mRNA expression upon CRISPR interference–based RIOK1 knockdown in PC3 cells. Data are expressed as means (95% CI). ∗∗∗∗P < 0.0001. [file mmc4.pdf]
